# The Wide Morphological Spectrum of Deep (Aggressive) Angiomyxoma of the Vulvo-Vaginal Region: A Clinicopathologic Study of 36 Cases, including Recurrent Tumors

**DOI:** 10.3390/diagnostics11081360

**Published:** 2021-07-28

**Authors:** Gaetano Magro, Giuseppe Angelico, Michal Michal, Giuseppe Broggi, Gian Franco Zannoni, Renato Covello, Stefano Marletta, Lucia Salvatorelli, Rosalba Parenti

**Affiliations:** 1 Department of Medical and Surgical Sciences and Advanced Technologies, “G. F. Ingrassia”, Anatomic Pathology, University of Catania, 95123 Catania, Italy; giuseppe.broggi@gmail.com (G.B.); lucia.salvatorelli@unict.it (L.S.); 2 Dipartimento Scienze della Salute della Donna, del Bambino e di Sanità Pubblica, Unità di Gineco-Patologia e Patologia Mammaria, Fondazione Policlinico Universitario A. Gemelli IRCCS, 00168 Rome, Italy; giuangel86@hotmail.it; 3 Department of Pathology, Charles University, Faculty of Medicine in Plzen, 30166 Plzen, Czech Republic; michal@fnplzen.cz; 4 Bioptical Laboratory, Ltd., 30166 Plzen, Czech Republic; 5 Unit of Gyneco-Pathology and Breast Pathology, Department of Women’s Health, Childhood and Public Health Sciences, A. Gemelli IRCCS University Hospital Foundation, Institute of Pathological Anatomy, Catholic University of the Sacred Heart, 00168 Rome, Italy; gianfranco.zannoni@unicatt.it; 6 Pathology Department, Regina Elena National Cancer Institute IRCCS, 00168 Rome, Italy; covello@ifo.it; 7 Department of Pathology and Diagnostics, University and Hospital Trust of Verona, 37010 Verona, Italy; stefano.marletta@gmail.com; 8 Department of Biomedical and Biotechnological Sciences (BIOMETEC), Section of Physiology, University of Catania, 95123 Catania, Italy; parenti@unict.it

**Keywords:** deep angiomyxoma, stromal tumors, lower female genital tract, immunohistochemistry, local recurrence

## Abstract

Background: Deep angiomyxoma (DAM) is currently included in the category of “specific stromal tumors of the lower female genital tract”, along with angiomyofibroblastoma, cellular angiofibroma and myofibroblastoma. Given the high rate of local recurrences, it is crucial to recognize DAM from other tumors that possess indolent behaviour. In the present paper, we analyzed the morphological and immunohistochemical features of 42 surgically-resected vulvo-vaginal DAMs (36 primary and 6 recurrent lesions) in order to widen the morphological spectrum of this uncommon tumor. Methods: A series of 36 cases of surgically-resected primary vulvo-vaginal DAMs were retrospectively collected. Locally recurrent tumors were also available for six of these cases. Results: Out of the primary tumors, 25 out of 36 exhibited the classic-type morphology of DAM. In the remaining cases (11/36 cases), the following uncommon features, which sometimes coexist with one another, were observed: (i) alternating myxoid and collagenized/fibrous areas; (ii) hypercellular areas; (iii) neurofibroma-like appearance; (iv) perivascular hyalinization; (v) microcystic/reticular stromal changes; (vi) “microvascular growth pattern”; (vii) perivascular cuffing; (viii) nodular leiomyomatous differentiation; (ix) hypocellular and fibro-sclerotic stroma. Among the six locally recurrent tumors the following features were observed: (i) classic-type morphology; (ii) hypocellular fibro-sclerotic stroma; (iii) extensive perivascular hyalinization, lumen obliteration and formation of confluent nodular sclerotic masses; (iv) hypercellularity. Immunohistochemically, the neoplastic cells of classic-type DAM in both primary and recurrent tumors were diffusely stained with desmin, suggesting a myofibroblastic nature; in contrast, the neoplastic cells showing elongated fibroblastic-like morphology and set in collagenized/fibrosclerotic stroma in both primary and recurrent tumors were negative or only focally stained with desmin, which is consistent with a fibroblastic profile. Conclusion: Although diagnosis of DAM is usually straightforward if typical morphology is encountered, diagnostic problems may arise when a pathologist is dealing with unusual morphological features, especially hypercellularity, extensive collagenous/fibrosclerotic stroma or neurofibroma-like appearance.

## 1. Introduction

Deep angiomyxoma (DAM) is a rare, locally infiltrative and non-metastasizing soft tissue tumor that usually arises in the vulvovaginal region, perineum and pelvis of adults [1,2,3,4,5]. Although DAM is most commonly found in women of reproductive age, sporadic cases have been reported in males, occurring almost exclusively in the genital area (scrotum, spermatic cord, inguinal region, perianal region and pelvic soft tissues) [6,7]. The reported age at presentation is 6–77 years, with the peak incidence occurring during the reproductive years. The female-to-male ratio is 6.6:1. DAM usually presents as a slow-growing, large-sized multilobular or polypoid mass with finger-like projections infiltrating the surrounding soft tissues [1,2,3,4,5]. The cut surface varies from an exclusively myxoid to a fibro-myxoid or predominantly fibrosclerotic tumor. Histologically, classic-type DAM is a paucicellular tumor composed of small-sized spindle-shaped to stellate cells set within a loose myxoid stroma containing numerous small-sized to medium-sized blood vessels [1,2,3,4,5]. Despite its bland-looking morphology, DAM exhibits an infiltrative growth and a significant risk of local recurrence. A wide local excision is difficult to achieve due to tumor infiltrative margins, which are often evident during the histological examination alone [8].

The present paper provides a detailed morphological and immunohistochemical study on a large series of primary and recurrent DAMs, emphasizing the uncommon morphological features that may cause diagnostic problems. Our immunohistochemical results suggest that the neoplastic cells of DAM are capable of modulating their differentiation from a myofibroblastic to a fibroblastic profile, resulting in a variable myxoid to fibrosclerotic stromal changes.

## 2. Materials and Methods

A series of 36 cases of surgically-resected vulvo-vaginal DAMs was retrospectively collected from the files of the University of Catania and the University of Pilsen. Locally recurrent tumors were also available for 6 of these 36 tumors. All patients were females and ranged in the ages from 43 to 65 years. Surgical specimens were submitted for histological examination in neutral-buffered 10% formalin, dehydrated using standard techniques, embedded in paraffin, 5 m sections cut and stained with hematoxylin and eosin (H&E). Immunohistochemical slides were available for each case and included the following antibody staining: vimentin (dilution: 1:40), desmin (dilution: 1:200), smooth muscle actin (dilution: 1:400), h-caldesmon (dilution: 1:100), CD34 (dilution: 1:400) and S100 (dilution: 1:300). The appropriate positive and negative controls were also available. The percentage of positively stained cells was assessed by semiquantitative optical analysis according to a 4-tiered system (1% positive cells: negative staining; 1–10% positive cells: focal staining; 11–50% positive cells: heterogeneous staining; >50% positive cells: diffuse staining). Staining intensity was graded as weak, moderate or strong intensity.

## 3. Results

Patients’ clinico-pathological features were retrieved from the original pathology reports. Upon gross examination, the majority of tumors presented as unencapsulated, poorly or vaguely circumscribed masses with a gelatinous, myxoid or fibrous cut surface. Tumor size was variable, ranging from 1.5 to 20 cm in the greatest dimension.

### 3.1. Primary Tumors

**Figure 1:**
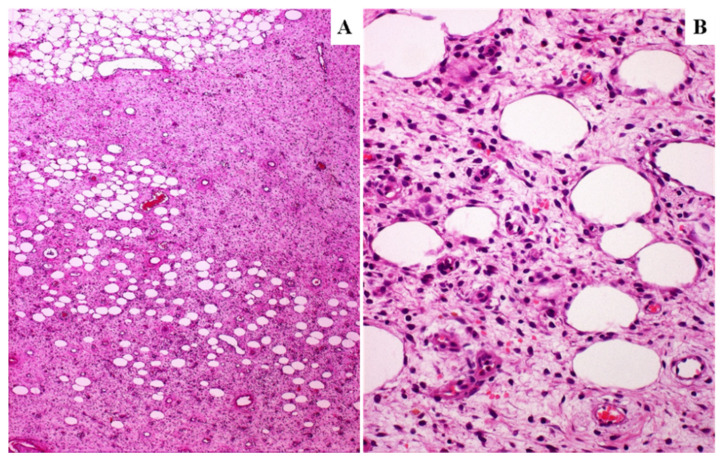
**Primary DAM.** Low (**A**) and high (**B**) power magnifications showing entrapment of adipose tissue.

All DAMs showed infiltrative margins with entrapment of adipose tissue and/or skeletal muscle (Figure 1A,B).

**Figure 2:**
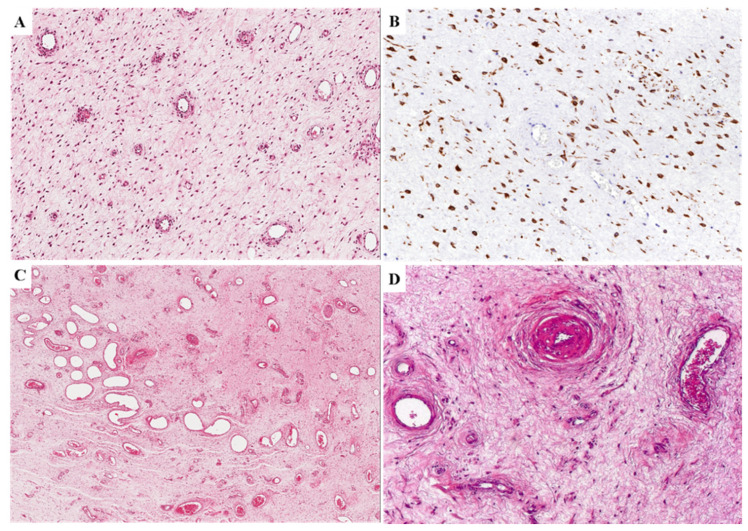
**Classic-type DAM**. (**A**) Hypocellular tumor composed of bland-looking small-sized spindled to stellate cells set haphazardly in an abundant myxo-edematous stroma with numerous capillary-like blood vessels. (**B**) Neoplastic cells of classic-type DAM are diffusely and strongly stained with desmin. (**C**) The vascular component ranges from capillary-like to medium/large-sized blood vessels. (**D**) The larger vessels occasionally contain a prominent smooth muscle layer.

Most tumors (25 out of 36) exhibited the classic-type morphology as previously reported in the literature, namely uniformly hypocellular tumor composed of bland-looking small-sized spindled to stellate cells set haphazardly in an abundant myxoedematous stroma rich in fine collagen fibrils and with a prominent vascular component (Figure 2A). The neoplastic cells exhibited poorly-defined scant cytoplasm and round to ovoid hyperchromatic nuclei with occasional small nucleoli. Mitoses were absent or rare (up to 1 mitosis × 10 HPF). Atypical mitoses, nuclear pleomorphism and necrosis were not observed. The neoplastic cells of classic-type DAM were diffusely (>50%) and strongly stained with desmin (Figure 2B) and only focally for smooth muscle actin and CD34 in a minority of cases (8 and 6 out of 25 cases, respectively). The vascular component ranged from capillary-like to medium/large-sized blood vessels (Figure 2A,C). The larger vessels often contained a prominent smooth muscle layer (Figure 2D).

**Figure 3:**
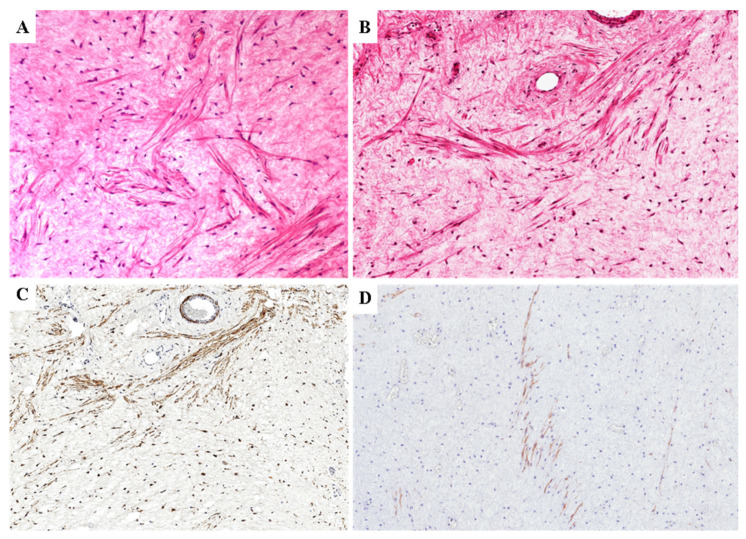
**Smooth muscle cell differentiaton in classic-type primary DAM**. (**A**) Isolated or small bundles of thin spindle-shaped cells with eosinophilic cytoplasm, which are interpreted as stromal cells with smooth muscle cell differentiation, are haphazardly scattered within the myxofibrillary stroma. (**B**) Occasionally, these spindle cells are found in proximity of blood vessels; however, an origin from the smooth muscle cell layer of the blood vessels cannot not be demonstrated. (**C**) Similar to the neoplastic stromal cells, even those with smooth muscle differentiation are stained with desmin. (**D**) Only stromal cells with smooth muscle differentiation are stained with h-caldesmon.

In 10 cases, as an additional finding, isolated or small bundles of thin spindle-shaped cells with eosinophilic cytoplasm interpreted as cells with smooth muscle differentiation were haphazardly scattered within the myxo-edematous stroma (Figure 3A,B); occasionally, they were in the proximity of blood vessels but the origin from the vascular smooth muscle layer could not be demonstrated (Figure 3A,B). These cells co-express desmin, smooth muscle actin and h-caldesmon (Figure 3C,D). No additional heterologous mesenchymal components were identified.

In the remaining cases (11 out of 36 cases), several uncommon features were observed. Notably, in all cases and at least focally, areas with the morphology of the classic-type DAM were identified and were crucial for a correct diagnostic interpretation. The following uncommon and sometimes co-existing features were observed (Table 1).

**Figure 4:**
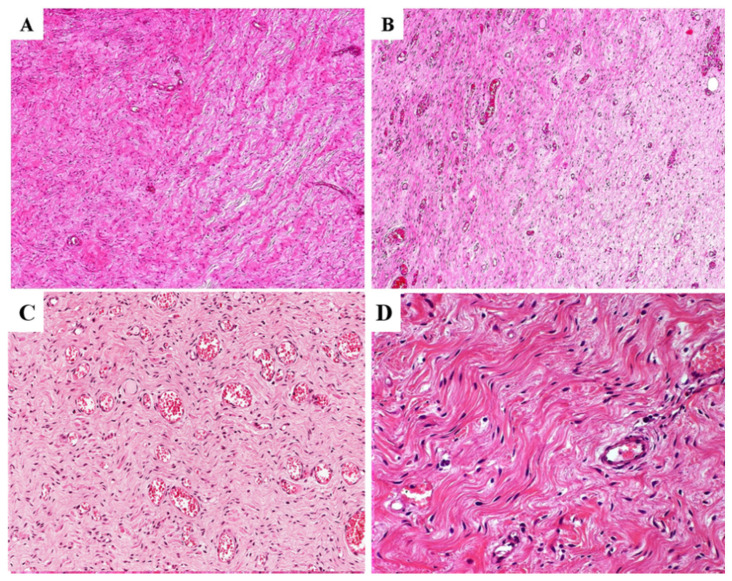
**Alternating myxoid to collagenized/fibrous stroma in primary DAMs**. (**A**,**B**) Two different tumors exhibiting alternating areas with myxoid and fibrous stroma (**C**,**D**). Two different tumors illustrating a myxofibrillary (**C**) or collagenized/fibrous stroma (**D**) with elongated fibroblastic-like spindled cells with wavy nuclei that are closely associated with thin and/or thick wavy eosinophilic collagen fibers. These tumor areas are closely reminiscent of a neurofibroma.

In (i) eight cases, alternating myxoid and collagenized/fibrous areas were observed (Figure 4A,B); four of these cases exhibited a neurofibroma-like appearance (up to 60% of the entire tumor), namely the neoplastic component was represented by elongated fibroblastic-like spindled cells with wavy nuclei that are closely associated with thin or thick wavy strands of eosinophilic collagen fibers (Figure 4C,D).

**Figure 5:**
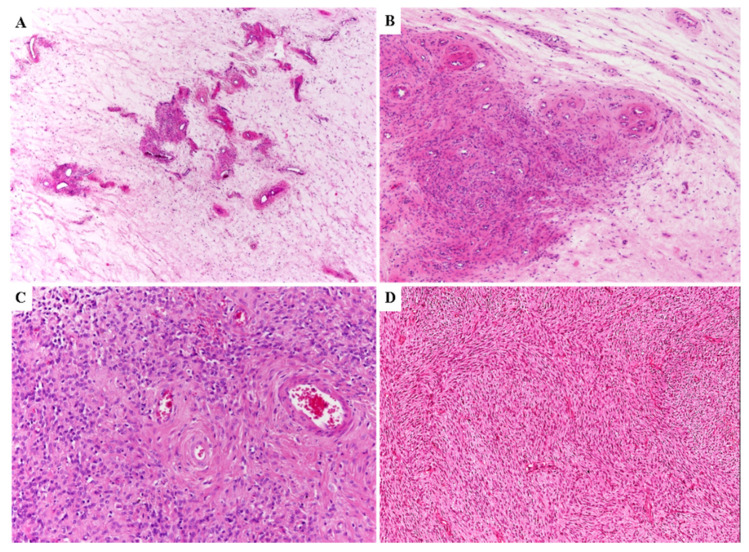
**Hypercellularity in 4 different primary DAMs**. (**A**,**B**) Hypercellularity is restricted to vascular regions or it has diffuse extension throughout the tumor (**C**,**D**). (**D**) In one tumor, neoplastic cells with fibroblastic-like morphology exhibited fascicular arrangement; notice the prominent vascular component.

In (ii) seven cases, hypercellular areas that account for 10 to 60% of the entire tumor are observed; although in most cases (five out of seven) hypercellularity was restricted to the perivascular regions (Figure 5A–C) and two tumors were diffusely hypercellular and composed predominantly of fibroblastic-like spindled cells with fascicular pattern (Figure 5D).

**Figure 6:**
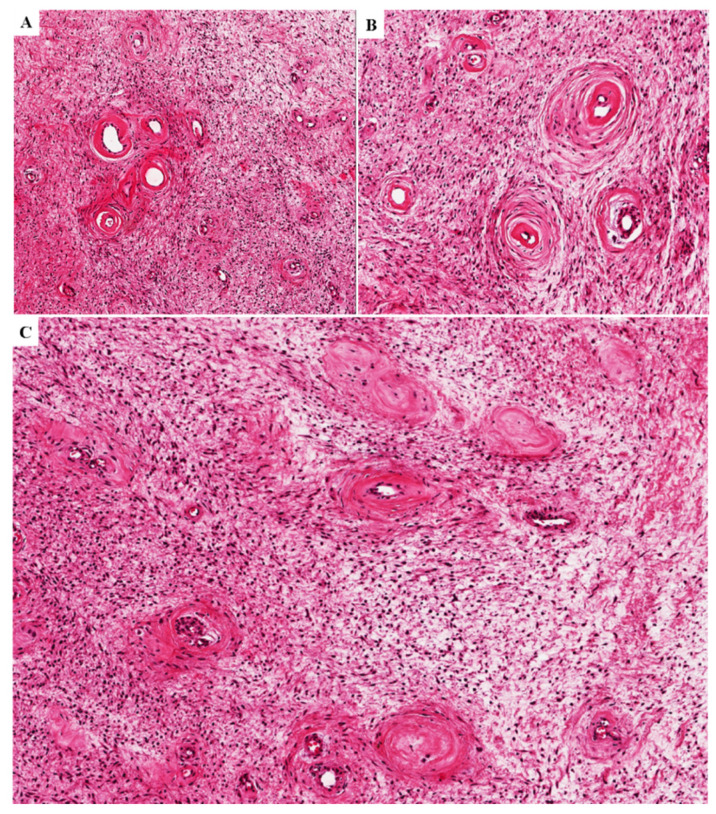
**Perivascular hyalinization in primary DAM**: (**A**) Small-sized and medium-sized blood vessels showing deposition of hyalinized eosinophilic collagen around the walls. (**B**) Perivascular cuffing of spindled cells with an onion-skin arrangement is also observed. (**C**) Some blood vessels showing fibro-sclerotic obliteration of the vascular lumens.

**Figure 7:**
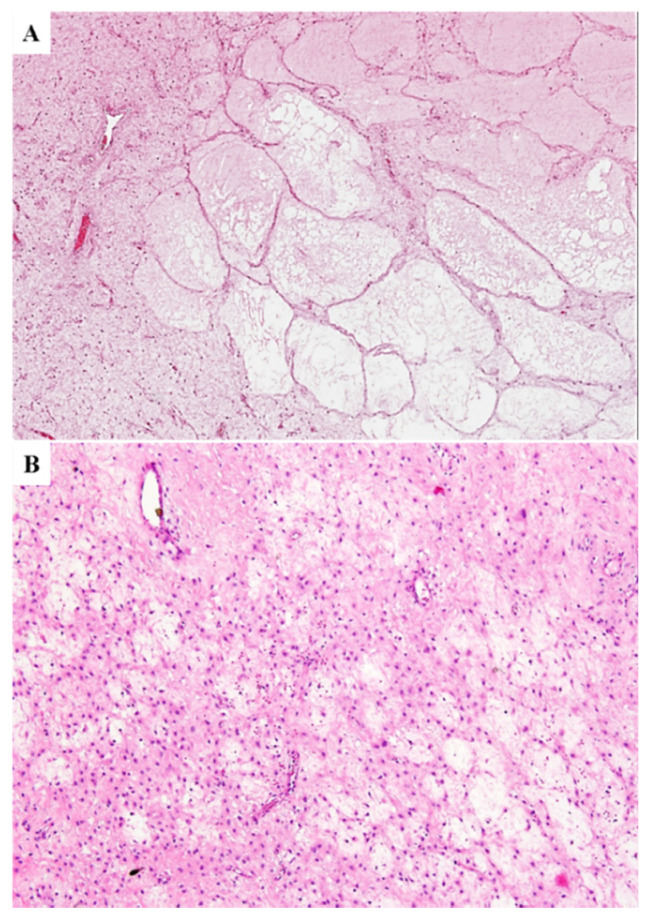
**Cystic stromal changes in primary DAM**. Abundant edematous stromal changes imparting a macrocystic (**A**) and microcystic (**B**) appearance to the tumor.

In (iii) four cases, perivascular hyalinization was observed (Figure 6A); as an additional finding, the perivascular cuffing of elongated neoplastic spindled cells (onion-skin arrangement) was identified in two cases (Figure 6B); fibro-sclerotic obliteration of the vascular lumens was observed in only in one case (Figure 6C);

In (iv) three cases, microcystic/reticular stromal changes were observed (Figure 7).

**Figure 8:**
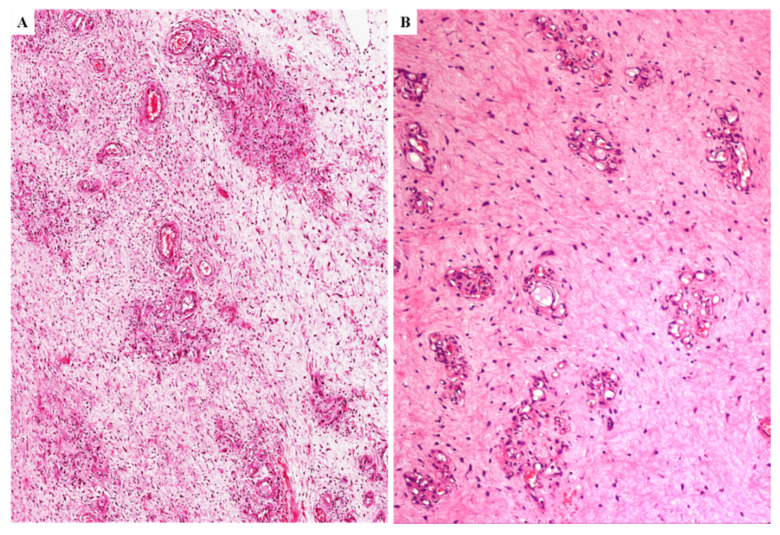
**Microvascular growth pattern in primary DAM**. Two different DAMs (**A**,**B**) in which the vascular component is mainly composed of closely packed capillary-sized blood vessels; this growth pattern is reminiscent of the so-called “*microvascular pattern*” as seen in glioblastoma.

In (v) two cases, the vascular component was mainly represented by a proliferation of capillary-like vessels closely packed with a “microvascular growth pattern” similar to that observed in glioblastoma (Figure 8).

**Figure 9:**
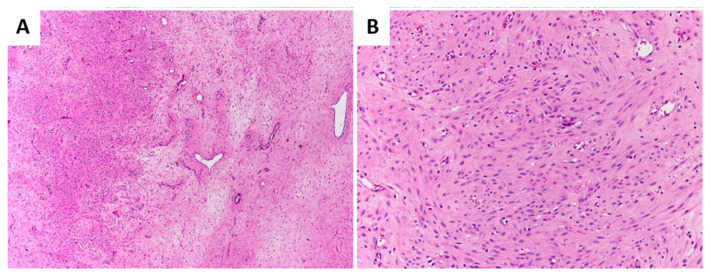
**Primary DAM with leiomyomatous nodular differentiation**. (**A**) In the context of an otherwise classic-type DAM, a vaguely nodular area composed of smooth muscle cells with focal fascicular arrangement is observed; (**B**) notice the morphological details of the cells.

In (vi) one case, there was a small-sized nodule (0.5 cm) composed of spindled cells with eosinophilic cytoplasm and ovoid nuclei containing one small nucleolus with the typical features of classic-type DAM; the cells, which are stained with vimentin, desmin, smooth muscle actin and h-caldesmon, were arranged in fascicles separated by scarce fibrous stroma and contained capillary-like blood vessels (Figure 9).

**Figure 10:**
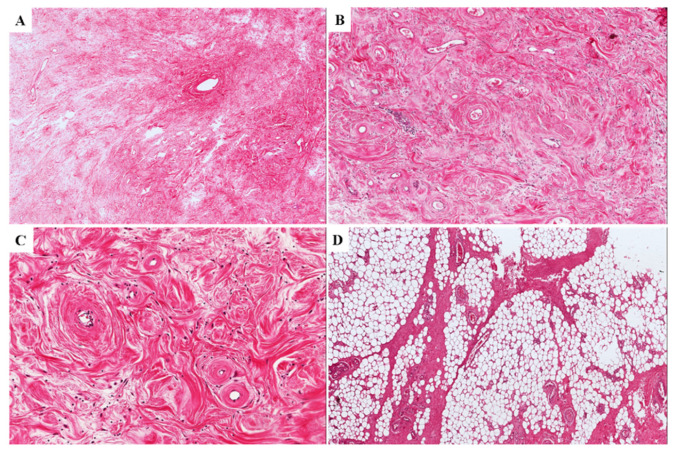
**Primary DAM with fibro-sclerotic stroma**. Low (**A**) and higher magnifications (**B**,**C**) illustrating a predominantly hypocellular and fibro-sclerotic stroma; thick keloid-like collagen bands (**B**) and blood vessels with perivascular hyalinization (**C**) are also observed; (**D**) this tumor shows a fibro-lipomatous appearance at the margins: Adipose tissue is diffusely infiltrated by fibrous septa.

This tumor was interpreted as a “classic-type DAM with nodular leiomyomatous differentiation”; (vii) in one case, tumor stroma was predominantly (>90%) hypocellular and fibro-sclerotic in nature; the tumor showed numerous thick keloid-like collagen bands and blood vessels with hyalinized walls (Figure 10A–C); the tumor exhibited a fibro-lipomatous appearance at the periphery (Figure 10D).

### 3.2. Locally Recurrent Tumors

Among the six cases of locally recurrent tumors, the following features were observed (Table 2).

**Figure 11:**
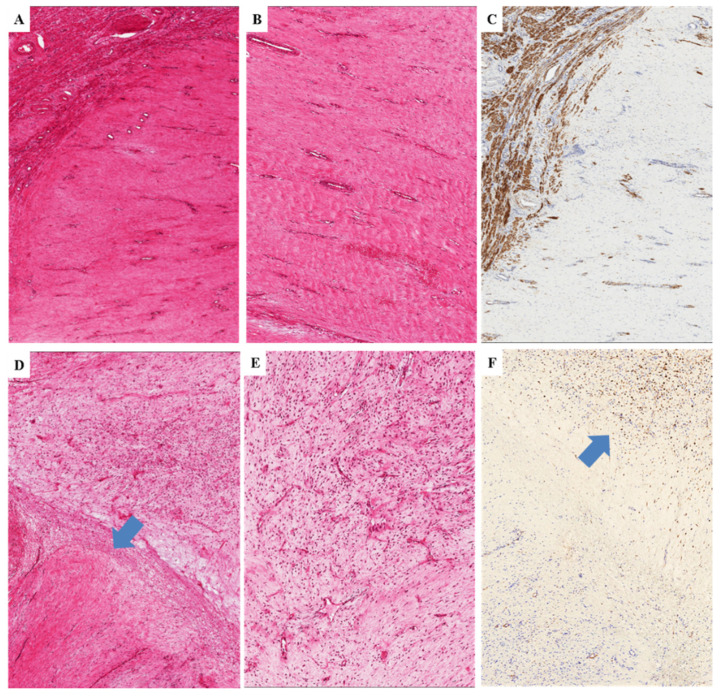
**Recurrent DAM**. Low (**A**) and higher magnifications (**B**,**C**) of a completely fibrosclerotic tumor infiltrating myometrium (M); (**C**) desmin expression is restricted to the smooth muscle cells of the myometrium. (**D**) Transition between fibrosclerotic tumor and area with the classic-type morphology of DAM (arrow). (**E**) The myxoid area contains numerous capillary-like vessels. (**F**) Only the neoplastic cells of the myxoid area are stained with desmin (arrow).

In (i) two cases, the tumors exhibited the same morphology of primary DAM, namely the classic-type features including hypocellular myxoid stroma and numerous small-sized to medium-sized blood vessels; in (ii) two cases, the tumors were predominantly (>95%) or exclusively hypocellular with abundant fibro-sclerotic stroma; in one case, the tumor was composed exclusively of thick keloid-like collagen bands with rarely interspersed spindle-shaped cells and medium-sized blood vessels with perivascular hyalinization; in one case, the tumor infiltrated the uterine myometrium as a large-sized uniformly collagenized lesion with rarely interspersed spindle-shaped cells and capillary-like blood-vessels (Figure 11A,B). The cells were negative or only focally positive for desmin (Figure 11C). Notably and only focally, a myxoid area with vascular component that is consistent with classic-type DAM was observed (Figure 11D,E); within this area, the neoplastic cells maintained the expression of desmin (Figure 11F). The primary counterpart of both tumor showed classic-type morphology.

**Figure 12:**
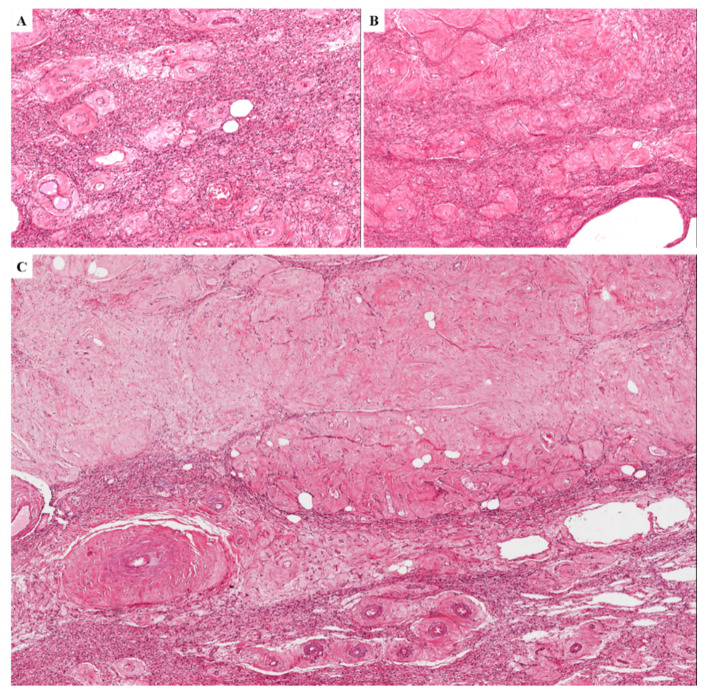
**Recurrent DAM**. (**A**,**B**) Tumor with lumen obliteration of the blood vessels; (**C**) the obliterated vessels form confluent nodular structures reminiscent of ovarian corpora albicantia.

In (iii) one case, the tumor showed alternating hypocellular and moderately cellular areas with numerous small-sized and medium-sized blood vessels demonstrating extensive perivascular hyalinization and lumen obliteration (Figure 12A–C); in some areas, the obliterated vessels formed confluent variably-sized fibrotic nodular structures closely resembling ovarian corpora albicantia (Figure 12C). The primary tumor counterpart exhibited the classic-type morphology with additional microcystic/reticular stromal changes.

In (iii) one case, the tumor demonstrated classic-type morphology of DAM, with the exception of hypercellular areas accounting approximately for 40% of the entire tumor; the primary counterpart exhibited the morphology of classic-type DAM.

### 3.3. Immunohistochemical Findings

The most relevant immunohistochemical findings encountered in our series have been summarized in Table 3 and compared with the largest DAMs series published in the literature.

## 4. Discussion

Among the tumors arising specifically from the stroma of the lower female genital tract, the recognition of DAM is crucial due to its risk of local recurrences [1,2,3,4,5]. The lastest edition of the *World Health Organization Classification of Tumors of Soft Tissue and Bone* [9] defines DAM as a uniformly paucicellular myxoedematous tumor with infiltrative margins and prominent vascular component. The present series, which comprises 36 primary vulvo-vaginal DAMs of which six recurrent cases were also available, shows that this unusual tumor encompasses a morphologic spectrum wider than originally described [1,2,3,4,5,9]. As previously reported for vulvo-vaginal cellular angiofibroma, angiomyofibroblastoma and myofibroblastoma [10,11,12], DAM may also exhibit morphologic variations in cellular composition, growth patterns, extracellular matrix and vascular components.

In our series, the classic-type morphology was found in 69.4% (25 out of 36 cases) of primary DAMs, while uncommon features were identified in the remaining 30.5% (11 out of 36 cases) of cases. We confirm that the isolated or small bundles of thin-sized eosinophilic stromal cells with smooth muscle differentiation profile (vimentin^+^/desmin^+^/-actin smooth muscle actin^+^/h-caldesmon^+^) can be identified in 27.7% (10 out of 36 cases) of primary DAMs. These cells had been previously labelled in the literature with the term “myoid bundles” [3,4]. In our opinion, these cells are not well-developed mature smooth muscle cells in that they lack the typical abundant deeply eosinophilic cytoplasm and cigar-shaped nuclei. As these cells have been found in proximity to the blood vessels and it has been suggested they arise from the smooth muscle layer of the larger tumor blood vessels [3,4]. However, in our series we encountered most of these cells away from the blood vessels, suggesting their metaplastic nature. We speculate that the neoplastic cells in a subset of primary DAMs may undergo leiomyomatous differentiation. The detection of a nodular stromal area of leiomyomatous differentiation in one case of our series seems to support this hypothesis. Accordingly, we suggest that the identification of a heterologous smooth muscle component in a vulvo-vaginal mesenchymal myxoid tumor favors the diagnosis of DAM. In the present study, we identified several uncommon morphological features in both primary and recurrent DAMS in terms of cell morphology, cellularity, stromal composition and vascularization. Although a primary DAM is usually a myxoid tumor, we found that there were alternating myxoid and more collagenized/fibrous areas in 22% of cases (8 out of 36 cases). Notably and in the collagenized/fibrous stroma, the neoplastic cells adopted an elongated fibroblastic-like appearance with undulating nuclei and imparted a neurofibroma-like morphology in four cases. To the best of our knowledge this neurofibroma-like pattern has not been previously emphasized in DAM and it could raise differential diagnostic problems. However, the lack of S100 expression and the identification, at least focally, of myxoid areas with the typical features of DAM are helpful in rendering correct diagnosis. Although it is believed that fibrosclerotic stroma is a feature of recurrent DAM, we encountered a single case of primary tumor with extensive stromal collagenization/sclerosis (>90% of the entire tumor), suggesting that DAM would be better regarded as “fibromyxoid” more than a “purely myxoid” tumor. As previously reported for other soft tissue myxoid tumors, such as myxoma, there is the possibility that DAM may also be, at least focally, hypercellular [13]. Although it is commonly believed that hypercellularity is relatively common in recurrent DAM [3,5,7,14,15], in our series it was found in approximately 19.4% (7 out of 36 cases) of primary DAMs. Despite this uncommon feature being mainly found around blood vessels, notably two cases were diffusely hypercellular with fascicular arrangements (50% and 60% of the entire tumor), which raises a concern for malignancy. The correct diagnosis was based on the identification of numerous variably-sized blood vessels and tumor areas exhibiting the classic-type morphology of DAM. Accordingly, the term “*hypercellular DAM*” seems to be appropriate for those tumors exhibiting diffuse hypercellularity.

Although it was originally believed that the hyalinized blood vessels were a characteristic feature of cellular angiofibroma among the specific mesenchymal tumors of the lower female genital tract, other studies demonstrated that they are not pathognomic since they were also observed in the vulvo-vaginal angiomyofibroblastoma and myofibroblastoma [10,11,12]. We encountered vessels with perivascular hyalinization in a minority of primary DAM (11% of cases: 4 out of 36 cases), suggesting that this morphologic finding is not helpful in the differential diagnosis. Notably and in two cases, a perivascular cuffing of the neoplastic cells with an onion-skin arrangement was observed. Another unusual finding relative to vasculature was the presence in two cases of a microvascular growth pattern similar to that observed in gliobastoma. To the best of our knowledge, this feature had not been previously reported in the literature. As far as recurrent DAMs are concerned, only a few studies, which are mostly based on single case reports or small series, have emphasized their morphological features. In this regard, recurrent DAMs are usually reported to exhibit foci of increased cellularity, nuclear pleomorphism, collagen deposition and increased vascularity [3,5,7,14,15]. In our series the fibrosclerotic stroma, including blood vessels hyalinization, was the most striking feature of recurrent DAMs. Rendering diagnosis of DAM in these cases is extremely difficult if pathologists are not aware of the primary diagnosis. However, we suggest that the possibility of DAM’s presence should be kept in mind when dealing with a fibro-sclerotic lesion that contains variably-sized blood vessels. In contrast, the hypercellularity, which is often cited as a typical feature of recurrent tumors [3,5,7,14,15], was found only one case of our series.

## 5. Conclusions

In conclusion, the present paper contributes to widen the morphological spectrum of primary and recurrent DAMs. Although the diagnosis of DAM is usually straightforward if typical morphology is encountered, diagnostic problems may arise when the pathologist is dealing with uncommon morphological features, especially for those primary or recurrent tumors exhibiting hypercellularity, extensive collagenous deposition with or without hyalinized blood vessels or neurofibroma-like appearance. The awareness of this possibility is crucial in order to avoid confusion with other mesenchymal lesions of the lower female genital tract. Finally, our study provides evidence that primary and recurrent DAM is a myofibroblastic/fibroblastic tumor with a variable myxoid to fibrosclerotic stroma. The classic-type DAM is predominantly a myxoid myofibroblastic tumor with vimentin^+^/desmin^+^/-smooth muscle actin^-^ profile. As previously suggested for mammary and extra-mammary myofibroblastoma [16], it is likely that the morphological variations in cellular (small spindled/stellate versus eloganted fibroblastic-like cells) and stromal composition (myxoid versus fibrosclerotic) reflect the plasticity of the neoplastic cells in adopting a myofibroblastic or fibroblastic profile (vimentin+/desmin-/-smooth muscle actin-) in response to various stimuli, including hormonal, genetic and microenviromental stimuli, that still remain to be elucidated. Based on our morphological and immunohistochemical observations, we suggest that the term *“deep angiofibromyxoma”* seems to be more appropriate rather than deep angiomyxoma. However, additional data on larger series of DAMs need to be collected in order to validate our hypothesis.

## Figures and Tables

**Table 1:** Primary DAMS with uncommon morphological features.

Number cases (%)	11/36 (30.5%)
Alternating myxoid/fibrous areas	8/11 (72.7%)
Hypercellular areas	7/11 (63.6%)
Neurofibroma-like areas	4/11 (36.3%)
Perivascular hyalinization	4/11 (36.3%)
Microcystic/reticular stromal changes	3/11 (27.7%)
Perivascular cuffing	2/11 (18.1%)
Microvascular growth pattern	2/11 (18.1%)
Nodular leiomyomatous differentiation	1/11 (9%)
Prominent fibrosclerotic stroma	1/11 (9%)

**Table 2:** Morphological features of recurrent DAMS.

Cases	Recurrent Tumor	Primary Tumor
Case n. 1	Classic-type morphology	Classic-type morphology
Case n. 2	Classic-type morphology	Classic-type morphology
Case n. 3	Hypocellular with prominent/exclusive fibrosclerotic stroma; Perivascular hyalinization	Classic-type morphology
Case n. 4	Hypocellular with prominent/exclusive fibrosclerotic stroma; Perivascular hyalinization	Classic-type morphology
Case n. 5	Hypocellular with prominent/exclusive fibrosclerotic stroma; Perivascular hyalinization with lumen obliteration and formation of confluent nodular structures	Classic-type morphology with microcystic/reticular stromal changes
Case n. 6	Hypercellularity (40% of tumor); Perivascular hyalinization with lumen obliteration	Classic-type morphology

**Table 3:** Comparative immunohistochemical findings in the largest reported series of primary DAMs.

References	Desmin	α-SMA	Vimentin	S-100	CD34	h-caldesmon
Steeper TA et al. [1]	NA	NA	NA	NA	NA	NA
Begin LR et al. [2]	NA	4/5 (80%)	NA	0/5 (0%)	NA	NA
Fetsch JF et al. [3]	22/22 (100%)	19/20 (95%)	17/17 (100%)	0/20 (0%)	8/16 (50%)	NA
Granter SR et al. [4]	13/14 (93%)	10/11 (91%)	NA	0/16 (0%)	NA	NA
Amezcua CA et al. [5]	8/11 (73%)	3/11 (27%)	11/11 (100%)	0/11 (0%)	NA	NA
Present series (Magro G et al.)	36/36 (100%)	10/36 (28%)	36/36 (100%)	0/36 (0%)	6/36 (17%)	10/36 (28%)

Abbreviations: DAM, deep angiomyxoma; -SMA, -smooth muscle actin; NA, not available.

## Data Availability

All data are available upon reasonable request to corresponding author.
